# Noncoding Centromeric RNA Expression Impairs Chromosome Stability in Human and Murine Stem Cells

**DOI:** 10.1155/2017/7506976

**Published:** 2017-07-11

**Authors:** David Y. L. Chan, Daniela Moralli, Suhail Khoja, Zoia L. Monaco

**Affiliations:** ^1^Wellcome Trust Centre for Human Genetics, Roosevelt Drive, Oxford OX3 7BN, UK; ^2^Special Block E, Reproductive medicine Unit, Department of O&G, The Chinese University of Hong Kong, Prince of Wales Hospital, Shatin, NT, Hong Kong; ^3^Biomedical Engineering, School of Engineering, 4 Colby Street, Medford, MA 02155-6013, USA

## Abstract

We analyzed the effect of transcribed noncoding RNA centromeric satellites on chromosome segregation in normal human and murine stem and fibrosarcoma cells. The overexpression of different centromeric alphoid DNAs in all cell lines induced a marked increase in chromosome mis-segregation in anaphase. Overexpression of centromeric mouse minor satellite also increased chromosome instability in the murine stem but not in human cells. Analysis of chromosome segregation in vivo showed disturbances in the mitotic progression, which was frequently unresolved. Live cell imaging revealed that overexpression of centromeric satellites resulted in several different chromosomal morphological errors in the cell nuclei. Our findings correlated with other reports that several centromeric noncoding RNAs are detected in different carcinoma cells and their expression resulted in segregation errors. Our study furnishes further insights into a novel source of genomic instability in human and murine cells. It has recently been shown that noncoding centromeric RNAs are present in some form of cancer, and thus, overexpression of several types of centromeric noncoding RNAs may be useful as a specific maker for neoplastic cells.

## 1. Introduction

Repetitive satellite DNA sequences (alphoid DNA) are essential for centromere formation and function during cell division [1, 2]. The centromere protein (CENP) requirements that affect chromosome function and segregation are complex [3]. Factors such as noncoding RNAs (ncRNAs) formed from transcripts of centromeric satellite DNA also influence chromosome and chromatin organisation in human [4] and murine [5, 6] cells. Centromeric RNAs are closely associated with centromeric chromatin and kinetochore formation. Furthermore, human centromeric RNAs were found to be transcribed in several tumour types but not in normal somatic tissues, suggesting that ncRNAs may play a role in cancer establishment or progression [7, 8]. In recent years, growing evidence has shown that transcription of noncoding RNA from pericentric and centromeric satellites could lead to mitotic or segregation errors [9]. The dosage balance of the ncRNAs is important for correct cell cycle progression, and balance perturbation might result in malignancy [10].

Human chromosome centromeres are comprised of tandemly repeated arrays of alpha (alphoid, *α*) satellite DNA arranged as 171 bp monomer units. The monomer arrays are chromosome specific but share a high homology in consensus sequence between chromosomes [11, 12]. The mouse centromere-specific minor satellite DNA is different in that the arrays are highly homogeneous between chromosomes. The *α* satellite DNA (with the exception of chromosome Y*α*) and the murine minor satellite contain a 17 bp motif known as the CENP-B box that binds to centromeric protein B (CENP-B) [13, 14]. In this study, we investigated the role of ncRNAs transcribed from different chromosome-specific centromeric satellites on chromosome segregation. In tumour and immortalised cells, chromosome segregation is impaired compared to that in normal cells. The overexpression of centromeric alphoid satellite DNA from chromosomes 17, 21, and Y in human stem (HUES-10) and fibrosarcoma (HT1080) cells resulted in a marked increase in chromosome mis-segregation events during anaphase. The analysis of HT1080 cells overexpressing 17 alphoid DNA during live cell imaging showed the disturbances in mitotic progression from metaphase to anaphase, which usually resulted in cell death. In comparison, the overexpression of the noncentromeric human DYZ1 satellite and the control vector DNA had no effect on chromosome segregation in HUES-10 and HT1080 cells. The minor satellite ncRNAs impaired chromosome segregation only in murine stem (ES) cells, indicating that the effect is species specific.

This study is the first example of using a live cell imaging system to observe the morphological deformities in the cell nuclei, resulting from centromeric ncRNA overexpression. The results highlight the importance of centromeric ncRNA expression on chromosomal instability.

## 2. Materials and Methods

### 2.1. Satellite Expression Constructs

Six eukaryotic expression vectors containing different repetitive DNA sequences were constructed, with the vector pIRESneo2 (pIneo2, Clontech), as the backbone. Add a sentence about generating the vector. A 2.7 kb fragment of core human chromosome 17 alpha satellite was obtained from EcoRI digestion of hBAC227J24 BAC (Kim et al., 1996) and cloned into the EcoRI site of pIneo2, to obtain pI-17*α*. Similarly, pI21*α* was assembled by cloning into pIneo2, a 1.4 kb fragment of human chromosome 21 alpha satellite, released by EcoRI digestion of pHSV21*α*HPRT-Neo (Moralli et al., 2006). The human Y chromosome alpha satellite was obtained by PCR amplification of total human genomic DNA using a specific primer (5′-ATG ATA GAA ACG GAA ATA TG-3′ and 5′-AGT AGA ATG CAA AGG GCT CC-3′. The 800 bp PCR product was cloned into an intermediate vector using the T/A cloning system (Promega, pGEM-Teasy system), excised by EcoRI digestion as an 850 bp fragment, and ligated into pIneo2 to obtain pIY*α*. The noncentromeric repetitive satellite DYZ1 from the human Y chromosome was amplified by PCR on genomic DNA using the primer sequences DYZ1 1F 5′-TCC CAT CCA ATC CAA TCT AC-3′ and DYZ1 1R 5′-GGA GTG GAA TAG ACA AGA GT-3′. As described for pIY*α*, the 1.4 kb DYZ1 PCR fragment was cloned into an intermediate vector, excised by EcoRI digestion as a 1.3 kb fragment, and ligated into pIRESneo2 to obtain the pIDYZ1 vector. A 1.7 kb fragment of mouse centromeric minor satellites was amplified from genomic mouse DNA with primers 5′-AAA AAA AAG GAT CCA AAA TTT AGA AAT GTC CAC TG-3′ and 5′-AAA AAA AAA GCT TAA GAT CTC CAT ATT TCA CGT CC-3′ and cloned into pBeloBAC 11 into BamH1 and BglII sites. The insert was then removed from the resulting vector (MNR) by NotI digestion and ligated into the pIneo2 vector into the NotI site, to generate pI-Minor. The pI-Major vector was similarly produced by PCR cloning of a 3.2 kb fragment with major satellite-specific primers 5′-AAA AAA AAG GAT CCG TGA GTT ACA CTG AAA AAC-3′ and 5′-AAA AAA AAA GCT TAA GAT CTT CCC GTT TCC AAC G-3′ on pBeloBAC 11. The fragment was then released by the E3.2 intermediate construct by NotI digestion and ligated into pIneo2. The specificity of all satellite sequences was tested by FISH on either human or mouse cells, to ascertain that they labelled specific chromosomes. The pH2BmCherry was produced by excising GFP from pH2BGFP using AgeI/NotI digestion; mCherry was cut from pmCherry (Clontech) by the same digestion. The mCherry fragment was ligated into the pH2B vector backbone to obtain pH2BmCherry.

### 2.2. Cell Culture and Transfection

Human fibrosarcoma HT1080 cells were grown in DMEM supplemented with 10% FCS and 1% penicillin/streptomycin. Mouse ES 14TG2A cells were grown in DMEM-GlutaMax with 15% fetal calf serum (FCS), 200 mM glutamine, 1× nonessential amino acids, 1% penicillin/streptomycin, 0.1 mM *β*-mercaptoethanol, and 1× leukemia inhibitory factor (Lif). Feeder-independent HUES-10 cells were grown on Matrigel (BD)-coated wells using the mTeSR medium (Stem Cell Technologies). TrypLE Express (Invitrogen) was used to enzymatically passage the hES cells. To increase single-cell survival, a ROCK inhibitor (Merck Biosciences) was added during each passaging step, at a final concentration of 10 *μ*M. All cells were incubated in a 37°C incubator supplied with 5% CO_2_. The day before transfection, 2 × 10^6^ cells were seeded in 6-well dishes. For each vector, 3 *μ*g of DNA was transfected into the target cells using 10 *μ*l of ExGen 500 transfecting agent per dish (Fermentas); the plate was centrifuged at 280*g* for 5 minutes. The cells were incubated at 37°C in an incubator supplied with 5% CO_2_.

### 2.3. Cell Fixation and Analysis of Segregation

After 72 hours, the cells were fixed for 10 minutes with 4% paraformaldehyde in PBS and counter-stained with DAPI. For each slide, 100 metaphases and anaphases were analyzed and the number and type of mis-segregation events scored. Each set of experiment was repeated at least three times. Fluorescence in situ hybridization (FISH) was carried out as described in (Moralli and Monaco, 2009). The slides were examined with an Olympus BX-51 epifluorescence microscope coupled to a JAI CVM4+ CCD camera. Images were acquired using Genus Software from CytoVision.

### 2.4. Immunostaining and FISH

Following transfection as outlined above, the cells were grown on glass slides and fixed in 4% paraformaldehyde. Immunofluorescence was performed using standard procedures with the following antibodies: mouse anti-Aurora B (BD, 1 : 100); mouse anti-H3 phospho-serine 10 (Upstate, 1 : 100); rabbit anti-H3 trimethyl-lysine 9 (Abcam, 1 : 100); and mouse anti-human CenpA (Abcam, 1 : 100). Fluorescence in situ hybridization (FISH) was carried out as described in Moralli and Monaco, 2009.

### 2.5. Noncoding RNA Expression Analysis

At 72 hours from transfection, total RNA was extracted from each cell line using the RNeasy kit (Qiagen), following the manufacturer's instructions. The RNA was treated with DNAse I (Qiagen) to remove contaminating DNA and reverse-transcribed into cDNA, using the RETROScript system (Ambion), with random decamer primers.

The quantification of 17 alpha overexpression in transformed cells was conducted by real-time PCR, using the PerfeCta SYBR Green Mix (Quanta Biosciences) on an iCycler machine (Bio-Rad) with the following primers: 17*α*Sat6F TTGTGGTTTGTGGTGGAAAA and 17*α*Sat6R CTCAAAGCGCTCCAAATCTC, and compared to that of a gene homogenously expressed in all cells (CENP B).

### 2.6. Live Cell Imaging

HT1080 cells were transfected with pH2BmCherry. Selection was applied with G418 at 300 *μ*g/ml, and a population stably expressing the transgene was recovered. The HT1080-H2BmCherry cells were transfected as outlined above with either pI-17a or pIneo2. After 48 hours, the chromosome segregation was analyzed by live cell imaging using a Zeiss LSM 510 confocal microscope for 48 hours. The multitracking function was used to avoid the bleed-through effect. Appropriate z-direction at 1-2 *μ*m for ten sections was captured on 63x oil objective for every 20 minutes for at least 12 hours.

## 3. Results

### 3.1. Noncoding Repetitive Satellite DNA-Expressing Vectors

**Figure 1:**
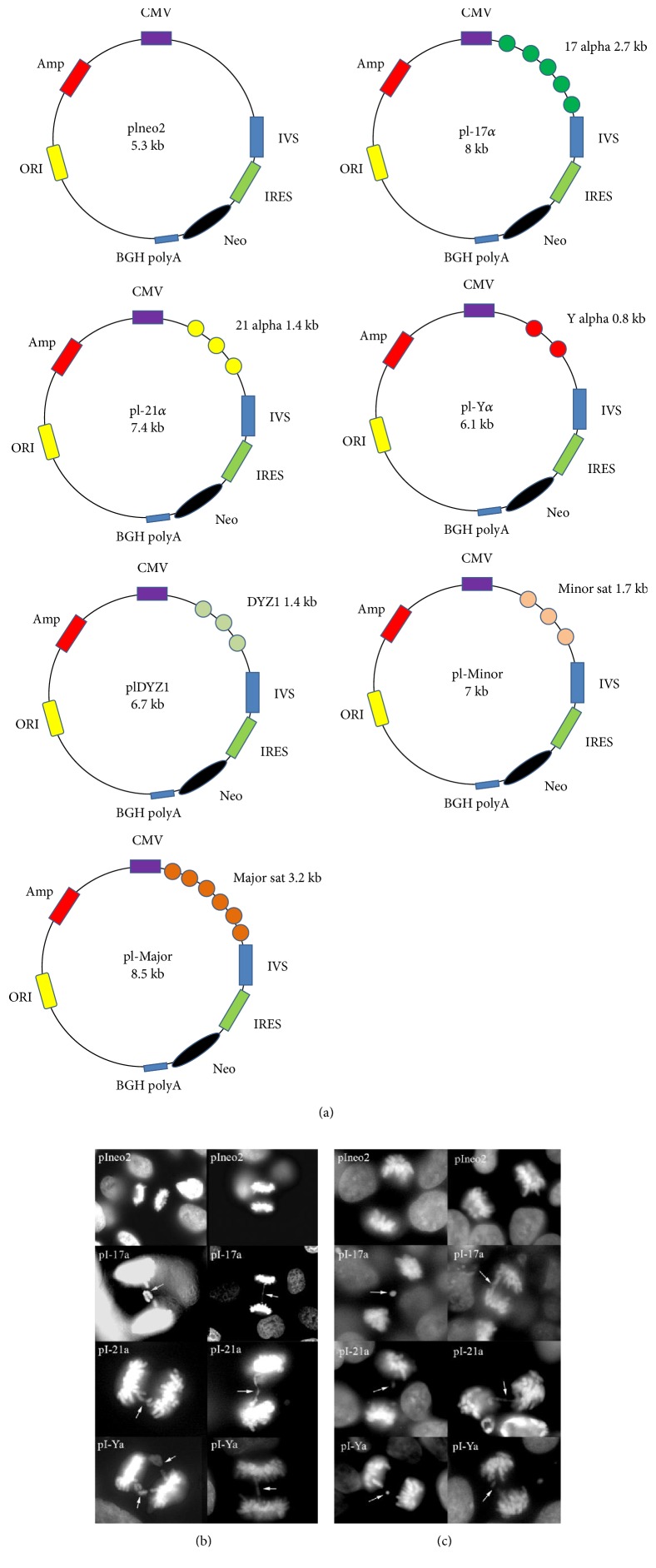
(a) Schematic vector maps. (b, c) Expression of 17*α*, 21*α*, and Y*α* induced mis-segregation in HT1080 (b) and HUES-10 (c) cells (white arrows).

To test the effect of centromere satellite expression on chromosome segregation, we constructed a series of expression vectors on an identical plasmid backbone, pIRESneo2 (pIneo2). The expression of all satellite inserts was initiated by the CMV promoter and the IRES sequence for the neo gene. Four of the vectors carried human or murine centromeric DNA: pI-17*α* (containing 2.7 kb of human *α* satellite DNA from chromosome 17), pI21*α* (containing 1.4 kb of *α* satellite DNA from chromosome 21), pIY*α* (containing 0.8 kb of *α* satellite DNA from chromosome Y), and pI-Minor (carrying 1.7 kb of mouse minor centromeric satellite). As control experiments, two more vectors containing noncentromeric satellite sequences were assembled: pIDYZ1 (carrying 1.1 kb of DYZ1 satellite from the human Y chromosome long arm) and pI-Major (containing 3.2 kb of mouse pericentromeric major satellite). The vectors are shown in Figure 1(a).

### 3.2. Expression of Centromeric Sequences Induces Mis-Segregation in Anaphase

The human (HUES-10, HT1080) and murine (E14TG2A) cells were transfected with the respective satellite expression constructs outlined above and the insert-less pIneo2 vector in a parallel control experiment. After 72 hours posttransfection, the cells were fixed and one hundred metaphase and anaphase cells on each slide were scored for segregation errors. The presence of delayed chromosome congression was scored as a metaphase error, while the presence of anaphase bridges or lagging chromosomes was scored as an anaphase error. At least three independent experiments were repeated for each vector and cell line, and the number of segregation errors observed for each satellite was compared to that of cells treated with the insert-less pISneo2 vector. The statistical significance of the differences was determined using the Student *t*-test for independent samples. The results are showed in Table 1 and Figures 1(b) and 1(c).

We further focussed on investigating the pI-17*α*-transfected cells. Real-time PCR was performed to confirm 17*α* RNA expression level in three different replicate experiments. On average, following transduction with pI-17*α*, we found that the centromeric satellites were transcribed at levels comparable to the single-copy control gene. The pI-17*α* expression levels correlated positively with the frequency of anaphase aberrations (*p* = 0.005): the higher the levels of the centromeric noncoding RNAs, the higher the percentage of cells that showed bridges and/or lagging chromosomes.

In HUES-10 and HT10180 cells, the overexpression of the 17*α*, 21*α*, and Y*α* human centromeric vectors did not affect the number of chromosomes involved in metaphase congression delay or the number of multipolar spindle cells (data not shown). However, approximately 20% of the anaphase cells analyzed after transfection with each chromosome *α* vector contained bridging or lagging chromosomes in HT1080 and HUES-10 cells (Figures 1(b) and 1(c)). No significant differences were observed between 17*α*, 21*α*, and Y*α*, in either HT1080 or HUES-10. The overexpression of noncentromeric satellite DYZ1 and pIneo2 vectors had no apparent effect on chromosome behaviour, in either metaphase or anaphase cells. The expression of mouse centromeric minor and major satellite DNA showed no significant effect on either human cell line (Table 1).

To determine if the chromosome 17 was preferentially involved in segregation errors when the 17*α* satellite was overexpressed, we conducted FISH in HT1080 cells, following transduction with pI-17*α* or control vectors, using a chromosome 17 whole-chromosome paint. No difference in chromosome 17 segregation efficiency was detected between cells transformed with pI-17*α* and pIneo2 (data not shown).

In mouse ES cells, none of the human satellites had any detectable effect on murine chromosome segregation. Overexpression of murine minor but not of major satellite DNA resulted in anaphase segregation errors (data no shown) compared to that of the pIneo2 control.

### 3.3. Immunohistostaining

The effect of centromeric ncRNAs on chromosome structure and segregation was analyzed by staining with specific antibodies for proteins or histone modifications involved in the kinetochore formation and the control of cell cycle progression. The localization of Aurora-B, CENP-C, CENP-A, histone H3 phosphorylated in serine 10, and H3 trimethylated in lysine 9 was investigated in HT1080 cells expressing the 17*α* and pIRESneo vector DNA. No difference was observed in localization or abundance of each protein in HT1080 cells overexpressing 17*α* and pIRESneo2 when compared to that in untreated cells (data not shown).

### 3.4. Live Cell Imaging on 17*α*-Expressing HT1080 Cells

**Figure 2:**
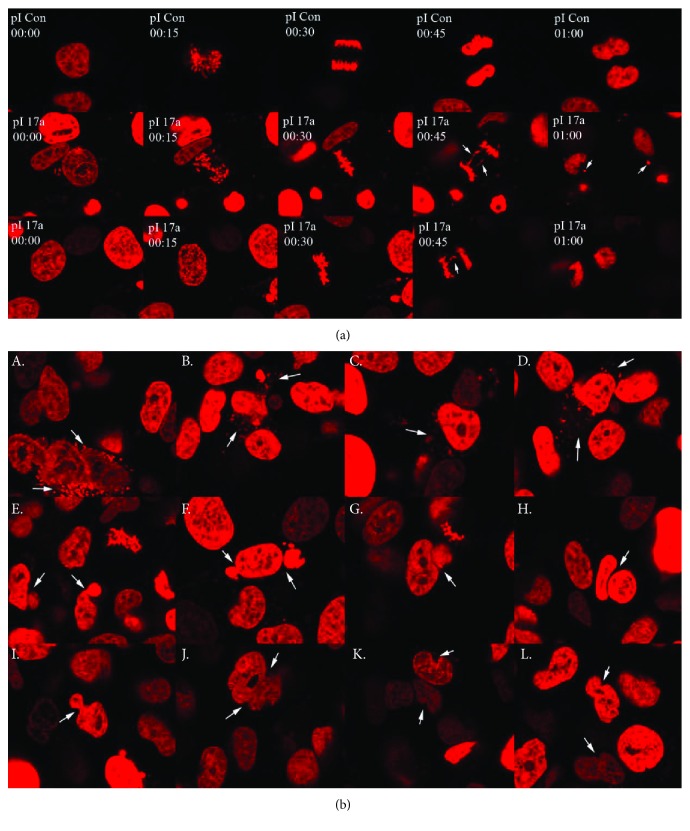
Live cell imaging on 17*α*-expressing H2B-mCherry-HT1080 cells. The pIneo2 controls did not show any mis-segregation ((a), top, left to right). Expression of 17*α* satellite in HT1080 induced mis-segregation events ((a), middle and bottom, left to right). Time lapse images were taken every 15-minute interval, and the image prior to mitosis was set at time zero. There were several interesting phenotypic effects discovered in 17*α*-expressing HT1080 cells, stars ((b), A–D), binucleated cells (E–H), and polylobed nuclei (I–L).

To visualize the chromosomal segregation events, HT1080 cells were initially transfected with a construct expressing a fusion gene between the H2B histone and the mCherry protein, which when incorporated into the chromatin rendered the chromosome a bright fluorescent red. The H2B-mCherry HT1080 cells were then transfected with pI-17*α* and pIneo2 as a control to observe chromosome segregation. Seventy-two hours after transfection, the cells were continuously analyzed by live cell imaging with a confocal microscope for 48 hours. In HT1080 cells transfected with pI-17*α*, 78 mitotic cells were captured and 33 (42.3%) showed bridging or lagging chromosomes in anaphase (Figure 2). In contrast, none of the H2B-mCHerry HT1080 cells transfected with pIneo2 showed bridging or lagging chromosomes in anaphase (data not shown).

The time lapse analysis clearly showed the presence of a strong red signal, scattered around the nuclei in a “star” phenotype. Nearly 40% of cells observed displayed this phenotype, while 14% of cells appeared binucleated, and 23% of cells had polylobed nuclei (Figure 2 and Table 2).

## 4. Discussion

In this report, we showed that overexpression of centromeric sequences from different suprachromosomal families induced a similar effect on chromosome segregation in both human stem (HUES-10) and fibrosarcoma (HT1080) cells. The 21*α* array includes a dimeric high-order repeat (HOR) belonging to the group of suprachromosomal families 1, 2, and 5; the 17*α* array has a pentameric HOR and belongs to suprachromosomal family 3, and the Y*α* array lacks a definable monomeric HOR and belongs to family 4 [15]. All of the alphoid suprachromosomal families except for family 4 contain the CENP-B box, a 17 bp motif that serves as a binding site for the centromeric protein B (CENP-B). However, overexpression of Y*α* in HUES-10 and HT1080 showed the same effect on chromosome segregation in both cell lines. These experiments clearly demonstrated that all human centromeric sequences tested affected segregation events in human tumour-derived and normal cells. This work is similar to a report by Bouzinba-Segard et al., whereby mouse minor satellite RNA expression and accumulation impaired mouse centromeric architecture and function [6].

Wong et al. recently showed that centromeric alphoid RNA is a key component for the assembly of nucleoproteins at the centromere and nucleolus [4], and this process may be disrupted from overexpression of ncRNAs. A more recent report showed that knocking down alpha satellite expression impaired chromosome segregation [16]. These findings are in line with our results, showing that noncoding satellite RNAs play an intriguing role to allow proper segregation to occur. It has been reported by Frescas et al. that KDM2A plays an important role in repressing centromeric satellite repeats; however, the specificity of the repression on chromosomal centromeric repeats was not further narrowed down [17].

Live cell imaging using HT1080-H2B-mCherry cells indicated that the expression of 17*α* RNA promoted mis-segregation in anaphase in real time detected with fluorescent histone H2B. Several phenotypic morphologies were observed. The cells overexpressing 17*α* RNA also revealed a high frequency of several other phenotypic events including binucleated (13.8%) and polylobed nuclei (23.2%) and “stars” (39.7%). Further work will be required to colocalize the noncoding RNA with the affected nuclei. The observations imply that centromeric RNA may be essential to maintain correct progression through mitosis through yet unclear mechanisms. Aberrant nuclear morphology is a feature associated with many forms of cancer cells and their subtypes [18, 19], and it has been speculated that nuclear morphological discrepancies may result from genomic instability.

There are growing evidences that various proteins interact with ncRNAs including CENP-A [20], polymerase II [9], heat shock protein [21], and aurora B [22], for their regulatory effects on chromosomal function and stability. However, none of the previous studies analyzed the effect of chromosome-specific repeats. RNA transcripts from centromeric and pericentromeric repetitive sequences have been identified in different organisms from yeast [23] to human [24], but the underlying mechanism has never been identified. Recent studies showed that alpha satellite nascent RNA is involved in heterochromatin modification. In addition, noncoding centromeric RNAs closely acted on the chromatin condensation or decondensation levels through the transcription of ncRNAs [25]; it is possible that our findings in this study are caused by the disruption of this finely tuned balance. The housekeeping expression level of ncRNA has never been studied, but it was suggested that the expression level could be affected by external factors such as temperature and chemical stress. Perturbation of the chromatin status elicited by ncRNAs is not surprising when related to the pathological status of the organism, as there were reports showing that elevation of ncRNA is involved in several types of cancers [8, 26–29].

Our study shows that the chromosome-specific alpha satellite RNAs affect the segregation of all chromosomes, as well as nuclear morphology. Further experiments are required to understand how centromeric ncRNAs affect chromosome segregation. This would include investigating the relationship between the ncRNA expression level and the severity of segregation errors incurred and understanding the downstream mechanism involved in centromeric RNA effects. However, our data suggest that evaluation of the levels of centromeric RNA expression and specific alterations in nuclear cell morphology could represent a useful cancer biomarker for some tumours.

## Figures and Tables

**Table 1:** The effects of noncoding centromeric RNAs on anaphase abnormalities.

Expression vectors and cell types	Anaphase abnormalities (%); number of error/total events	Significant level	Standard deviation and standard error
HT 1080 cells			
Control pIneo2	9.7 (136/1400)	NA	3.36/0.93
pI-17*α*	21.3 (170/800)	<0.0001	7.11/2.51
pI-21*α*	21.3 (128/600)	<0.0001	5.17/2.11
pI-Y*α*	25.3 (76/300)	<0.0001	1.15/0.66
pI-DYZ1	7.5 (90/1200)	Not significant	2.90/0.83
pI-Major	10.6 (74/700)	Not significant	3.34/1.26
pI-Minor	13.6 (95/700)	Not significant	3.47/1.37
HUES-10 cells			
Control pIneo2	5.0 (15/300)	NA	4.58/2.65
pI-17*α*	18.7 (56/300)	<0.02	6.51/3.76
pI-21*α*	19.7 (59/300)	<0.029	8.5/4.91
pI-Y*α*	16.3 (49/300)	<0.014	1.15/0.67
pI-DYZ1	6.7 (20/300)	Not significant	3.79/2.19
pI-Major	6.3 (19/300)	Not significant	4.93/2.85
pI-Minor	5.7 (17/300)	Not significant	7.23/4.18
mES cells			
Control pIneo2	4.7 (14/300)	NA	2.52/1.45
pI-17*α*	6.0 (18/300)	Not significant	2.00/1.15
pI-21*α*	6.0 (18/300)	Not significant	2.00/1.15
pI-Y*α*	7.3 (22/300)	Not significant	0.58/0.33
pI-DYZ1	6.0 (18/300)	Not significant	3.61/2.08
pI-Major	7.3 (22/300)	Not significant	2.89/1.67
pI-Minor	14.3 (43/300)	<0.025	5.51/3.18

**Table 2:** The effects of alpha 17 satellite expression in the HT1080 cell.

Alpha 17 satellite expression in HT1080	
Types of abnormalities	% of abnormality
Anaphase abnormality	42.3
Delayed metaphase	9
Polylobed nuclei	23.2
Binuclei	13.8
Stars	39.7
